# Can subordinate performance simultaneously reduce leader ostracism and promote leader recognition? A moderated mediation model

**DOI:** 10.3389/fpsyg.2025.1588034

**Published:** 2025-05-09

**Authors:** Qin Xu, Hao Huang, Xinran Zhang, Shuming Zhao, Lulu Zhou

**Affiliations:** ^1^School of Economics & Management, Southeast University, Nanjing, China; ^2^School of Labor and Human Resources, Renmin University of China, Beijing, China; ^3^School of Business, Nanjing University, Nanjing, China

**Keywords:** subordinate performance, subordinate contribution, leader ostracism, leader recognition, outcome dependence on subordinate

## Abstract

In recent years, the positive influences of leadership on subordinate performance have been extensively studied. However, whether high-performing subordinates can, in turn, change the way leaders lead them remains underexplored. Based on social exchange theory, this research examines the mediating role of subordinate contribution in the relationship between subordinate performance and leader ostracism and recognition, as well as the moderating role of the leader’s outcome dependence on subordinate. Results from a multi-wave and multi-source field survey comprising 245 subordinates and 68 leaders indicate that subordinate performance increases subordinate contribution, which in turn, reduces leader ostracism and promotes leader recognition. Moreover, outcome dependence on subordinate reinforces the positive impact of subordinate performance on subordinate contribution, and the mediating effect of subordinate contribution. These findings not only provide a theoretical explanation of how and under what conditions subordinate performance can be welcomed by the leader, but also offer valuable insights for organizations to mitigate negative leader responses and foster positive ones.

## 1 Introduction

Employee performance serves as the cornerstone of organizational success, acting as the vital bridge between strategic objectives and operational achievement (Decramer et al., 2013; Sarwar and Muhammad, 2021; Ni et al., 2022). In the era of VUCA (i.e., volatile, uncertain, complex, ambiguous), where businesses operate in a highly competitive and rapidly changing environment (Bennett and Lemoine, 2014; Garavan et al., 2024), the ability to sustain high employee performance has evolved from a competitive advantage to a survival imperative. It directly impacts productivity, innovation, customer satisfaction, and ultimately, the company’s profitability (Gong et al., 2009; Call and Ployhart, 2021). Consequently, extant studies have predominantly focused on identifying factors that can improve employee performance, with leadership consistently considered an important category of antecedents (e.g., Tsai et al., 2009; Wadei et al., 2023; Umrani et al., 2024).

However, can employees who achieve high performance, in turn, alter their leaders’ behavioral patterns? Scholars have conducted preliminary explorations of this question. For example, subordinate performance has been found to decrease negative leadership behaviors (e.g., abusive supervision, Tariq and Weng, 2018; Lyubykh et al., 2022) and promote positive leadership behaviors (e.g., empowering leadership, Cao et al., 2022; Wang et al., 2022). Nevertheless, examining these two effects in isolation fails to fully capture how high-performing subordinates may receive preferential treatment from leaders, necessitating an integrated investigation. Given that subordinates generally seek to avoid leader ostracism (Fiset and Boies, 2018; Azeem et al., 2024) and desire leader recognition (Wayne et al., 2002; Malek et al., 2020), this study investigates whether subordinate performance can simultaneously reduce leader ostracism and increase leader recognition.

To explain how subordinate performance influences leadership behavior, existing studies have drawn on theories like social comparison theory (Tariq et al., 2021), affective events theory (Li et al., 2024) and self-control framework (Liang et al., 2016). However, these studies have principally focused on the mediating role of leader affect in the relationship between subordinate performance and leadership behavior, while neglecting the crucial role of leader cognition. Social exchange theory posits that individual interactions are guided by the principle of reciprocity (Emerson, 1976; Cropanzano et al., 2017; Akkermans et al., 2024), individuals weigh potential outcomes before deciding whether to engage in specific relationships or behaviors. As mentioned earlier, subordinate performance exerts a positive effect on the individual, the team, and the organization (Gong et al., 2009; Call and Ployhart, 2021), directly or indirectly supporting leaders’ work. Consequently, leaders tend to foster good relationships with subordinates rather than ostracize them and may reciprocate by recognizing subordinates’ contributions. Based on the above line of reasoning, we suggest that subordinate performance may reduce ostracism and enhance recognition through leader-perceived subordinate contribution.

Furthermore, leader personal factors (e.g., leader social orientation, Khan et al., 2018; Tariq et al., 2021) and leader-subordinate dyadic factors (e.g., leader-subordinate guanxi, Duarte et al., 1994; Cao et al., 2022) are acknowledged as two significant types of boundary conditions in the process by which subordinate performance influences the leader. Indeed, the nature of leaders’ tasks affects their interpretations of subordinates’ performance (Xu and Li, 2024). In team tasks requiring a close collaboration between leaders and subordinates, subordinates’ proficiency in completing assigned tasks can influence goal attainment. Therefore, it is necessary to integrate the roles of both parties into the process of how subordinate performance influences leadership. A potential factor is the leader’s outcome dependence on subordinate, defined as the extent to which subordinates’ efforts impact leaders’ work outcomes (Walter et al., 2015). Specifically, when outcome dependence on subordinate is high, the fulfillment of the leader’s goals is tied to the competence and dedication of the subordinate (de Jong et al., 2007). In this case, the contribution of subordinate performance to the leader is magnified, and its impact on leader ostracism and recognition is subsequently enhanced. Therefore, we maintain that outcome dependence on subordinate may play a moderating role in the above relationship.

**Figure 1:**
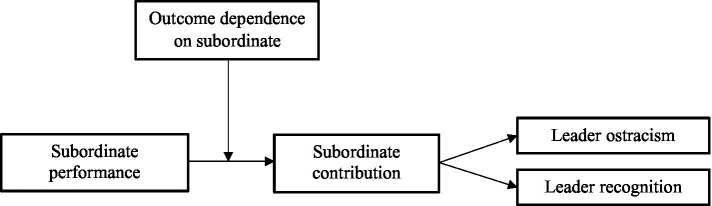
Research model.

Overall, based on social exchange theory, we develop a research model as shown in Figure 1. By exploring the mediating role of subordinate contribution between subordinate performance, leader ostracism and recognition, as well as the moderating role of outcome dependence on subordinate, there are three main theoretical contributions expected from this study. First, we integrate the inhibitory or promotional effects of subordinate performance on leadership behaviors, which contributes to a more comprehensive understanding of the positive influences of subordinate performance on leadership. Second, we investigate the leader’s cognitive mechanisms between subordinate performance and leadership behavior based on social exchange theory, offering a new perspective to explain how subordinate performance impacts leadership behavior. Finally, we incorporate the moderating role of leader personal factors and leader-subordinate dyadic factors in the process of subordinate performance influencing leadership, which facilitates a deeper understanding of the conditions under which subordinate performance can be welcomed by the leader.

## 2 Theoretical background and hypotheses

### 2.1 Subordinate performance and contribution

As one of the groups with significant responsibility for the development of the organization, leaders tend to evaluate subordinate performance positively, a factor that can facilitate the efficient functioning, goal accomplishment and competitive advantage of the organization (Yang and Wei, 2017; Hulshof et al., 2020; Van Scotter and Van Scotter, 2021). For example, leaders may believe that high performing subordinates are high in conscientiousness (Lyubykh et al., 2022), beneficial (Mawritz et al., 2017) and trustworthy (Cao et al., 2022). Similarly, we argue that subordinate performance can increase the contribution to the leader, reflecting a subordinate’s assistance to a leader in accomplishing team goals (Spreitzer, 1995). It is important to distinguish these two constructs: subordinate performance refers to behaviors or outcomes mandated by job descriptions (Williams and Anderson, 1991), whereas completing basic duties does not necessarily translate into contributions to the leader’s work. For instance, in an organization that promotes innovation, subordinates who strictly adhere to established procedures may fail to generate incremental value for their leaders.

Subordinate performance can lead to higher contribution for the following reasons. High-performing subordinates tend to be better able to complete work tasks, solve problems, innovate, and move projects forward (Chun et al., 2018; Alaybek et al., 2022), all of which are important indicators for leaders to assess their subordinates’ contributions. In addition, such subordinates usually have more interactions with their leaders (Lam et al., 2007; Rivera et al., 2024), and accordingly, leaders are more likely to notice their accomplishments. Since leaders typically evaluate the contributions of subordinates based on their performance (Breuer et al., 2013; Wang et al., 2023), when a subordinate demonstrates efficient and high-quality work results, it not only facilitates the achievement of team goals but also adds value to the organization, then the leader may perceive the subordinate as a greater contributor. Thus, the following hypothesis is formulated.

*H1*. Subordinate performance is positively related to contribution.

### 2.2 Subordinate contribution and leader ostracism

In general, leaders do not display negative leadership behaviors toward subordinates who have made valuable contributions. For instance, subordinates who are good at their jobs are not prone to passive leadership (Krasikova et al., 2013), and more specifically, help from subordinates can reduce a leader’s laissez-faire leadership behavior (Lee et al., 2024). Besides, proactive subordinates are less likely to be derailed during their career development (Crossley et al., 2023). Following this logic, we assume that subordinate contributions decrease leader ostracism, defined as leaders’ exclusion or marginalization of subordinates from decision-making processes, group activities, or social interactions within an organization (Jahanzeb et al., 2018; Hsiao et al., 2024).

Within an organization, leaders often welcome members who exert a positive influence, and enhance efficiency and effectiveness (Crouch and Yetton, 1988; Zhang et al., 2024). When subordinates make substantial contributions through active work attitudes, efficient task execution, and innovative thinking or specialized skills, they alleviate leaders’ workloads and assist leaders in better achieving their goals (Tang et al., 2008; Zhang et al., 2020). This, in turn, indirectly improves leaders’ management effectiveness (Brewer et al., 1994; Vasquez et al., 2021). Therefore, such subordinates are not only less likely to experience leader ostracism, but also more likely to become key members whom leaders rely on and value. Accordingly, we propose the following hypothesis.

*H2a*. Subordinate contribution is negatively related to leader ostracism.

### 2.3 Subordinate contribution and leader recognition

In a similar vein, subordinates who make outstanding contributions tend to be treated well by their leaders. For example, leaders exhibit greater amiability toward subordinates with frequent task-related interactions (Crouch and Yetton, 1988). Subordinates with effective communication skills are more likely to be included in leaders’ affairs (Abson and Schofield, 2022). Moreover, leaders are inclined to seek advice from subordinates when perceiving alignment with their goals (Liu and Dong, 2020). Correspondingly, we believe that subordinate contribution can facilitate leader recognition, leaders’ acknowledgment or appreciation of subordinates’ work, capabilities, and attitudes (Wayne et al., 2002; Malek et al., 2020).

Generally, leaders are concerned with their own outcomes and benefits (Porter et al., 2016; Mai et al., 2022). When subordinates’ work can improve efficiency, enhance customer satisfaction, advance the success of projects and bring economic gains (Lichtenthaler and Fischbach, 2018; Huang et al., 2024), such contributions are naturally noticed and appreciated by leaders. To acknowledge and motivate subordinates (Miao et al., 2013; Zhang and Min, 2021), leaders may recognize their efforts through verbal praise or other means. Therefore, the following hypothesis is proposed.

*H2b*. Subordinate contribution is positively related to leader recognition.

### 2.4 The mediating role of subordinate contribution

Combining hypotheses 1 and 2a/2b, subordinate contribution may mediate the relationship between subordinate performance and leader ostracism and recognition. Social exchange theory states that there are mutually beneficial exchange relationships between individuals (Emerson, 1976; Cropanzano et al., 2017; Akkermans et al., 2024). When one person provides a benefit to another, the recipient experiences a psychological obligation to reciprocate. In organizational context, subordinates with high performance deliver positive work outcomes and potential organizational rewards to leaders. For results-driven leaders, such subordinates are indispensable to the attainment of their goals; thus, leaders tend to maintain and strengthen the relationships with them rather than ostracize them. Furthermore, leaders would reciprocate by acknowledging subordinates’ contributions. Taken together, we formulate the following hypotheses.

*H3a*. Subordinate contribution mediates the relationship between subordinate performance and leader ostracism.

*H3b*. Subordinate contribution mediates the relationship between subordinate performance and leader recognition.

### 2.5 The moderating role of outcome dependence on subordinate

Further, previous research suggests that leaders’ interpretations of subordinate performance are not fixed, but can be moderated by leader personal factors (e.g., leader social orientation, Khan et al., 2018; Tariq et al., 2021) and leader-subordinate dyadic factors (e.g., leader-subordinate guanxi, Duarte et al., 1994; Cao et al., 2022). However, few studies have integrated these two types of boundary conditions. According to social exchange theory (Emerson, 1976), the development of interpersonal relationships or engagement in specific behaviors is contingent upon the presence of tangible benefits. For leaders, the significance of subordinate performance to them changes with their degree of reliance on subordinates. To address this gap, we introduce outcome dependence on subordinate (i.e., the extent to which a leader’ performance is reliant on the action and outcome of a subordinate, Walter et al., 2015; Xu and Li, 2024), and explore its moderating role in the relationship between subordinate performance and contribution.

When leaders’ outcome dependence on subordinates is high, they heavily rely on subordinates to achieve desired results and goals (Shi et al., 2013; Xu and Li, 2024). High-quality subordinate performance leads leaders to focus intently on outcomes and perceive such subordinates as critical contributors to goal attainment. Conversely, low-performing subordinates often fail to complete tasks successfully. When leaders heavily depend on these subordinates, they are more likely to notice employees’ shortcomings and ultimately perceive them as poor contributors to team goals. Thus, the positive relationship between subordinate performance and leader-perceived contribution is stronger when leaders’ outcome dependence on subordinate is high.

When outcome dependence on subordinate is low, subordinates play a less critical role in leaders’ goal attainment. On one hand, even if subordinates excel at their tasks, leaders may not strongly recognize the outstanding performance of the subordinate (Moss and Martinko, 1998; Li et al., 2024) because of the weak connection between the leader and the subordinate. On the other hand, although low-performing subordinates may cause problems like delayed task or substandard quality, leaders who do not depend too much on subordinates are unlikely to perceive these problems as threatening team goal achievement. Therefore, the positive relationship between subordinate performance and leader-perceived contribution is weaker when leaders’ outcome dependence on subordinates is low. Taken together, the following hypothesis is developed.

*H4*. Outcome dependence on subordinate strengthens the relationship between subordinate performance and contribution.

### 2.6 Moderated mediation

Synthesizing hypotheses 3a, 3b, and 4, it is possible that outcome dependence on subordinate moderates the mediating role of subordinate contribution in the relationship between subordinate performance and leader ostracism and recognition. In the case of high outcome dependence on subordinate, subordinate performance makes a significant contribution and becomes indispensable to the leader’s realization of team goals. Consequently, leaders are less likely to ostracize such subordinate. In addition, leaders are more likely to grant recognition to subordinates for prominent contributions. On the contrary, when outcome dependence on subordinate is low, the impact of subordinate performance on the leader’s objectives and outcomes is minimal. As such, leaders may perceive little need to ostracize under-performing subordinates or benefit from recognizing high-performing ones. In summary, we develop the following hypotheses.

*H5a*. Outcome dependence on subordinate strengthens the mediating effect of subordinate contribution on the relationship between subordinate performance and leader ostracism.

*H5b*. Outcome dependence on subordinate strengthens the mediating effect of subordinate contribution on the relationship between subordinate performance and leader recognition.

## 3 Method

### 3.1 Sample and procedure

In this study, subordinates and their leaders from a company in a southeastern city of China were invited to participate in a questionnaire survey. Before the study commenced, the research team explained its purpose, emphasized the voluntary nature of participation, and promised that their personal information would be kept strictly confidential and that the data would be used only for academic analysis. Based on the practice of previous studies (Rank et al., 2009; Cai et al., 2023), data collection was divided into two periods, with a one-month interval, aiming to improve the causality between variables and mitigate common method variance.

In the first session, data of subordinate performance and outcome dependence on subordinate were collected, both filled out by leaders toward each subordinate, and demographic information was collected for subordinates and leaders. With the help of human resources manager, participants’ questionnaires were numbered and then sent to 290 subordinates and 72 leaders. After eliminating invalid ones with incomplete or mismatched responses, 278 subordinate data and 71 leader data were reserved. In the second period, leaders rated subordinates’ contribution one by one, and subordinates were asked to evaluate leaders’ ostracism and recognition on them. After sorting, the valid data returned this time included 245 subordinates and 68 leaders. The overall effective recovery rate of the two surveys was 84.48% for subordinate data and 94.44% for leader data. Table 1 described the demographic information of subordinates and leaders. For the subordinates, their average age was 37.24 years old and 38.00% of them were male. Among the sample of leaders, the average age was 42.41 years old and men accounted for 48.50%.

**Table 1:** Sample characteristics.

Variable	Category	Subordinate sample		Leader sample	
		Frequency	Percentage	Frequency	Percentage
Age	21–25 years old	16	6.50%		
	26–30 years old	51	20.80%	4	5.90%
	31–35 years old	49	20.00%	9	13.20%
	36–40 years old	52	21.30%	19	28.00%
	41–45 years old	32	13.10%	13	19.10%
	46–50 years old	17	6.90%	9	13.20%
	51–55 years old	16	6.50%	10	14.70%
	56–60 years old	12	4.90%	4	5.90%
Gender	Male	93	38.00%	33	48.50%
	Female	152	62.00%	35	51.50%

### 3.2 Measures

All the scales used in the study were originally developed in English and the standard procedure of “translation and back-translation” was carried out. The 7-point Likert scale was adopted for all main variables.

#### 3.2.1 Subordinate performance

To measure subordinate performance, three items (1 = “far below average”; 7 = “far above average”) were selected from Williams and Anderson’s (1991) seven-item scale and supplemented with the subject. An example is “This subordinate meets formal performance requirements of the job” (*α* = 0.969).

#### 3.2.2 Subordinate contribution

We adapted the wording of the three items (1 = “strongly disagree”; 7 = “strongly agree”) developed by Spreitzer (1995) to make them applicable to leaders evaluating the contributions of subordinates, with a sample “This subordinate makes me feel self-assured about my capabilities to perform my work activities” (*α* = 0.976).

#### 3.2.3 Leader ostracism

We adapted the wording of the six items (1 = “do not remember”; 7 = “often”) developed by O’Reilly et al. (2015) to make them applicable to subordinates evaluating leader ostracism. An example is “My leader excludes me from influential roles or committee assignments” (*α* = 0.980).

#### 3.2.4 Leader recognition

Similar to leader ostracism, we adapted the wording of the three items (1 = “strongly disagree”; 7 = “strongly agree”) developed by Wayne et al. (2002) to measure leader recognition, with a sample “My leader recognizes me” (*α* = 0.828).

#### 3.2.5 Outcome dependence on subordinate

To measure outcome dependence on subordinate, we converted the two items (1 = “strongly disagree”; 7 = “strongly agree”) developed by Wee et al. (2017) into declarative sentences. An example is “I am dependent on this subordinate for materials, means, information, etc.” (*α* = 0.800).

#### 3.2.6 Control variables

Referring to previous studies (e.g., Ferris et al., 2019; Jahanzeb et al., 2021), we controlled for subordinates’ and leaders’ age (1 = “21–25 years old”; 2 = “26–30 years old”; 3 = “31–35 years old”; 4 = “36–40 years old”; 5 = “41–45 years old”; 6 = “46–50 years old”; 7 = “51–55 years old”; 8 = “56–60 years old”) and gender (1 = “male”; 2 = “female”).

## 4 Results

### 4.1 Confirmatory factor analyses

The hypothetical five-factor model was tested, and its main indexes were: *χ*^2^ = 145.604, df = 109, *χ*^2^/df = 1.336, RMSEA = 0.037, CFI = 0.993, TLI = 0.991, showing a good fit to the data. According to the features of the variables, we set up four competition models, namely four-factor model (subordinate contribution and outcome dependence on subordinate combined), three-factor model (subordinate contribution and outcome dependence on subordinate combined; subordinate performance and leader recognition combined), two-factor model (variables except leader ostracism combined) and one-factor model (all variables combined), as shown in Table 2. Comparative analysis found that the original model fitted better, indicating that the five variables in this study had good discriminative validity.

**Table 2:** Confirmatory factor analyses.

Competition model	*χ*^2^	df	*χ*^2^/df	RMSEA	CFI	TLI
Four-factor model	565.404	113	5.004	0.128	0.914	0.896
Three-factor model	1565.508	116	13.496	0.226	0.724	0.677
Two-factor model	1891.084	118	16.026	0.248	0.663	0.611
One-factor model	2987.695	119	25.107	0.314	0.455	0.377

### 4.2 Descriptive statistics and correlations

Table 3 exhibited the means, standard deviations and correlations of variables. From the table, we could see that, subordinate performance was significantly positively correlated with contribution (*r* = 0.439, *p* < 0.01). Subordinate contribution was significantly negatively related to leader ostracism (*r* = −0.170, *p* < 0.01) and positively associated with leader recognition (*r* = 0.156, *p* < 0.05). The above results preliminarily supported H1 and H2a-b.

**Table 3:** Descriptive statistics and correlations.

Variable	M	SD	1	2	3	4	5	6	7
Subordinate variables									
Subordinate age	3.85	1.84							
Subordinate gender	1.62	0.49	−0.244^**^						
Subordinate performance	5.67	1.08	0.060	0.027					
Subordinate contribution	5.38	1.18	−0.016	0.154^*^	0.439^**^				
Leader ostracism	2.28	1.10	0.173^**^	−0.098	−0.094	−0.170^**^			
Leader recognition	5.49	1.00	−0.049	−0.050	0.064	0.156^*^	−0.312^**^		
Outcome dependence on subordinate	4.68	1.38	−0.041	0.140^*^	0.432^**^	0.361^**^	−0.155^*^	−0.018	
Leader variables									
Leader age	4.88	1.62							
Leader gender	1.51	0.50	−0.164						

*N* (subordinates) = 245; *N* (leaders) = 68. ^*^p < 0.05 and ^**^p < 0.01.

### 4.3 Hypotheses testing

As subordinates were partially nested within leaders, we used HLM (i.e., hierarchical linear modeling) to test our hypotheses. The results of regression analyses were presented in Table 4. As shown in Model 1, subordinate performance was positively related to contribution (*γ* = 0.375, *p* < 0.001), supporting H1. Consistent with H2a-b, subordinate contribution significantly decreased leader ostracism (*γ* = −0.159, *p* < 0.01, Model 3) and increased leader recognition (*γ* = 0.159, *p* < 0.01, Model 4). To test the mediating role of subordinate contribution between subordinate performance and leader ostracism (H3a)/recognition (H3b), bootstrap method was used for analysis, the number of repeated samplings was set to 5,000 and the confidence interval level was 95%, the results provided support for H3a [indirect effect = −0.071, 95% CI = (−0.161, −0.001)] and H3b [indirect effect = 0.066, 95% CI = (0.006, 0.135)].

**Table 4:** Regression analyses of HLM.

Predictors	Subordinate contribution		Leader ostracism	Leader recognition
	Model 1	Model 2	Model 3	Model 4
Intercept	3.646^***^	5.697^***^	3.182^***^	5.106^***^
Leader age	−0.106	−0.097	−0.041	−0.028
Leader gender	−0.066	−0.087	−0.087	0.087
Subordinate age	0.007	0.011	0.106^**^	−0.037
Subordinate gender	0.158	0.149	−0.063	−0.191
Subordinate performance	0.375^***^	0.381^***^		
Subordinate contribution			−0.159^**^	0.159^**^
Outcome dependence on subordinate		0.193^*^		
Subordinate performance × outcome dependence on subordinate		0.153^**^		

*N* (subordinates) = 245; *N* (leaders) = 68. ^*^p < 0.05, ^**^p < 0.01, and ^***^p < 0.001.

**Figure 2:**
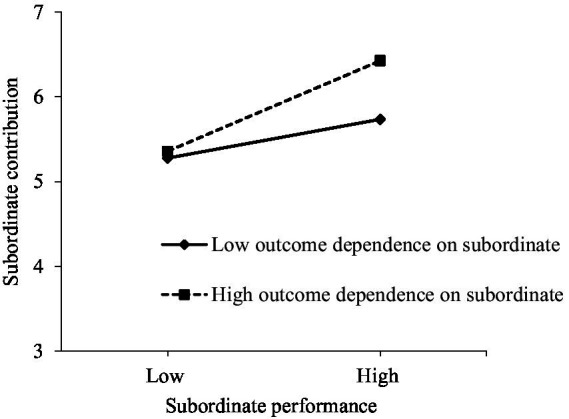
Moderating effects of outcome dependence on subordinate.

According to Model 2, the interaction term involving subordinate performance and outcome dependence on subordinate significantly predicted subordinate contribution (*γ* = 0.153, *p* < 0.01), which supported H4. To demonstrate the moderating effects of outcome dependence, we drew a simple slope plot (see Figure 2). When outcome dependence on subordinate was high, subordinate performance had stronger positive effect on contribution (*γ* = 0.534, *p* < 0.001). In the case of low outcome dependence on subordinate, such effect was weakened (*γ* = 0.229, *p* < 0.01).

Further, the moderated indirect effect of outcome dependence on subordinate was examined. The results displayed in Table 5 revealed that, the indirect influence of subordinate performance on leader ostracism through subordinate contribution was not significant at a low level of outcome dependence on subordinate [indirect effect = −0.033, 95% CI = (−0.086, 0.001)] but remained significant when outcome dependence on subordinate was high [indirect effect = −0.096, 95% CI = (−0.218, −0.002)], besides, the difference between the two situations was significant [indirect effect = −0.064, 95% CI = (−0.159, −0.001)]. In addition, when outcome dependence on subordinate was low, the indirect effect of subordinate performance on leader recognition through subordinate contribution was not significant [indirect effect = 0.030, 95% CI = (−0.001, 0.074)] but kept significant under high outcome dependence on subordinate [indirect effect = 0.089, 95% CI = (0.007, 0.192)], and the difference between the two conditions was significant [indirect effect = 0.059, 95% CI = (0.003, 0.145)]. In summary, these results supported H5a-b.

**Table 5:** Moderated mediation results for outcome dependence on subordinate.

Dependent variable	Condition	Effect	LLCI	ULCI
Leader ostracism	Low (−1 SD)	−0.033	−0.086	0.001
	High (+1 SD)	−0.096	−0.218	−0.002
	Difference	−0.064	−0.159	−0.001
Leader recognition	Low (−1 SD)	0.030	−0.001	0.074
	High (+1 SD)	0.089	0.007	0.192
	Difference	0.059	0.003	0.145

## 5 Discussion

Based on social exchange theory, this study investigates the mediating role of subordinate contribution between subordinate performance and leader ostracism and recognition, as well as the moderating role of outcome dependence on subordinate. Empirical analyses of 245 subordinate data and 68 leader data indicate that subordinate performance reduces leader ostracism/promotes leader recognition by increasing subordinate contribution. Besides, the positive influence of subordinate performance on subordinate contribution and the mediating effect of subordinate contribution are stronger when outcome dependence on subordinate is high.

### 5.1 Theoretical implications

The above findings contribute to the existing literature in four primary ways. First, we integrate the effects of subordinate performance on leadership behaviors. Although prior research has demonstrated that subordinate performance can either inhibit negative leadership behaviors (e.g., abusive supervision, Tariq and Weng, 2018; Lyubykh et al., 2022) or promote positive leadership behaviors (e.g., empowering leadership, Cao et al., 2022; Wang et al., 2022), whether these two effects can coexist remains underexplored. Our results reveal that subordinate performance can not only reduce leader ostracism (a negative behavior), but also increases leader recognition (a positive behavior), thus providing a more comprehensive confirmation of subordinate performance’s dual impacts on leadership.

Secondly, this study investigates the antecedents of leader ostracism and recognition. Previous studies have emphasized leadership styles such as leader ostracism and recognition (e.g., Wayne et al., 2002; Huang and Yuan, 2024), but they have under-investigated the motivations behind why leaders ostracize or recognize subordinates. Addressing scholars’ calls to explore the antecedents of leadership behaviors (Yang et al., 2019; Cao et al., 2022), the findings of this study indicate that low subordinate performance leads to leader ostracism, whereas high subordinate performance fosters leader recognition. This framework advances understanding of the conditions under which leader ostracism and recognition emerge.

Third, the present research enriches the mechanisms by which subordinate performance influences leadership behavior. Prior studies have mainly used social comparison theory (Tariq et al., 2021), affective events theory (Li et al., 2024) and self-control framework (Liang et al., 2016) to explain why subordinate performance impacts leadership behavior from an affective perspective, yet they have underemphasized cognitive mechanisms. Based on social exchange theory, we introduce subordinate contribution as a mediating variable and examine its role in the relationships between subordinate performance, leader ostracism, and leader recognition. This study offers a novel cognitive lens for interpreting how subordinate performance shapes leadership behavior.

Lastly, we broaden the boundary conditions of the relationship between subordinate performance and leader behavioral responses. Although leader individual differences (e.g., leader social orientation, Khan et al., 2018; Tariq et al., 2021) and leader-subordinate dyadic factors (e.g., leader-subordinate guanxi, Duarte et al., 1994; Cao et al., 2022) were identified as two critical categories of moderators influencing leaders’ interpretations of subordinate performance, whether these two categories can be incorporated remains underexplored. Our results demonstrate that outcome dependence on subordinate enhances the effect of subordinate performance on leader ostracism and recognition via leader-perceived subordinate contribution, thus offering a deeper understanding of the conditions under which subordinate performance can be welcomed by leaders.

### 5.2 Practical implications

At the practice level, the results of this study have valuable implications for organizations seeking to mitigate negative leader responses and amplify positive ones. Firstly, high-performing subordinates are not only less likely to be ostracized by their leaders, but also able to receive more recognition from their leaders. Organizations should encourage employees to pursue high performance. For example, they should ensure that each employee is clear about his or her work objectives and expected outcomes (Kearney et al., 2019; Zhang et al., 2023). Moreover, in order to motivate employees, organizations must establish fair, transparent and timely reward systems (Mickel and Barron, 2008; Cao et al., 2022).

Additionally, subordinate contributions to team goals can reduce leader ostracism and increase their recognition, so organizations should facilitate leaders’ awareness of subordinates’ contributions. For instance, organizations should encourage leaders to collaborate with subordinates on projects to enhance interaction and cooperation (de Poel et al., 2012; Ali et al., 2022), thereby deepening leaders’ understandings of subordinates’ work and contributions. At the same time, organizations can provide leaders with special training courses (Lacerenza et al., 2017; Cohrs et al., 2020), such as emotional intelligence enhancement and communication skills strengthening, to help leaders better appreciate subordinates’ efforts.

Furthermore, when outcome dependence on subordinate is high, the indirect effects of subordinate performance on leader ostracism and recognition are both amplified. Thus, organizations can strengthen leader-subordinate job interdependence through optimized job design. For example, organizations should encourage leaders to delegate important tasks and projects to subordinates (Cheong et al., 2019; Tang et al., 2020). By assigning critical responsibilities that have a direct impact on team success, leaders become more reliant on their subordinates to achieve desired outcomes. Besides, organizations should implement evaluation systems where leaders’ performance reviews significantly incorporate subordinate development, engagement, and productivity (Walter et al., 2015; Hu et al., 2022).

### 5.3 Limitations and future research directions

It should be noted that this study has limitations. For instance, while leader-rated subordinate performance and subordinate-rated leadership (i.e., leader ostracism and recognition) collected at different time points effectively minimize common method variance (Rank et al., 2009; Cai et al., 2023), cross-sectional data cannot test causality among variables (Ugwu et al., 2016; Kanat-Maymon et al., 2020), particularly given that leadership is often regarded as an antecedent of subordinate performance (Chan, 2017; Ding and Yu, 2020). Longitudinal designs and experimental methods, which are better suited to infer causality (Wang et al., 2018; Moin et al., 2024) could be employed in future research to validate our model.

Although our research hypotheses are supported in China and subordinate performance may affect leaders in other cultural contexts (e.g., Khan et al., 2018; Lyubykh et al., 2022), the generalizability of these findings to other countries and regions remains unconfirmed. Future research could test our model in countries or regions other than China to explore whether subordinate performance universally reduces leader ostracism and promotes leader recognition.

Finally, this study has only focused on the effects of subordinate task performance on leader behaviors. Indeed, subordinate performance also includes extra-role performance (Landry and Vandenberghe, 2012; Vera and Sánchez-Cardona, 2023), with prior research demonstrating that subordinates’ constructive ideas (Xu et al., 2023) and personal initiative (Sharma and Kirkman, 2015) influence leadership behaviors. Investigating how subordinate extra-role performance affects leader ostracism and recognition would further refine our theoretical framework.

## 6 Conclusion

Previous studies have mainly focused on how leadership contributes to high subordinate performance. Applying the social exchange framework, we in turn explore how high-performing subordinates influence leadership style. When subordinates’ efforts are significant to the leader’s achievement of desired outcomes, their high performance can be positively evaluated by the leader, which in turn leads to less ostracism and more recognition from the leader. We hope that our findings will encourage future research to reveal more conditions conducive to reducing negative leadership behaviors and promoting positive ones.

## Data Availability

The original contributions presented in the study are included in the article/supplementary material, further inquiries can be directed to the corresponding author.
